# Where is the common ground between bone marrow mesenchymal stem/stromal cells from different donors and species?

**DOI:** 10.1186/s13287-015-0144-8

**Published:** 2015-08-18

**Authors:** Elena Jones, Richard Schäfer

**Affiliations:** Leeds Institute of Rheumatic and Musculoskeletal Medicine, Leeds University, Room 5.24 Clinical Sciences Building, St James’s University Hospital, Leeds, LS9 7TF UK; Institute for Transfusion Medicine and Immunohaematology, German Red Cross Blood Donor Service, Baden-Württemberg-Hessen gGmbH, Johann-Wolfgang-Goethe University Hospital, Sandhofstrasse 1, D-60528 Frankfurt am Main, Germany

## Abstract

Mesenchymal stem/stromal cells (MSCs) feature promising potential for cellular therapies, yet significant progress in development of MSC therapeutics and assays is hampered because of remarkable MSC heterogeneity in vivo and in vitro. This heterogeneity poses challenges for standardization of MSC characterization and potency assays as well as for MSC study comparability and manufacturing. This review discusses promising marker combinations for prospective MSC subpopulation enrichment and expansion, and reflects MSC phenotype changes due to environment and age. In order to address animal modelling in MSC biology, comparison of mouse and human MSC markers highlights current common ground of MSCs between species.

## Introduction

In contrast to hematopoietic stem cells (HSCs), progress in the field of mesenchymal stem/stromal cells (MSCs) has been impeded by inconsistency in terminology and the lack of suitable assays to test the self-renewal of cells in vivo [1]. Furthermore, academic and industrial efforts in the development of cell therapies with culture-expanded MSCs tend to surpass our basic-science understanding of the physiological roles of these cells in vivo [1–3]. It is fair to say that nearly 15 years since a seminal letter by Stanton Gerson, MSCs remain to some degree ‘second class marrow citizens’ [4] in comparison with much better defined HSCs—which at first sight might appear surprising because advanced, and still improving, detection and characterization technologies have been available for both cell entities for decades. At second sight, however, major challenges prevail to reliably define characteristics and properties shared by MSCs derived from various donors and from different species. Besides phenotypic features obviously pertinent to species (e.g. mouse vs. human; see later), the heterogeneity of MSC preparations in vitro as well as the heterogeneous distribution of stromal cells in the bone marrow (BM) in vivo may be regarded as major impediments that significantly slow down progress in basic and translational MSC research as well as in development of MSC therapies.

Many published studies have described significant heterogeneity of cultured MSC preparations [5, 6]. These studies have identified the sources of such heterogeneity, amongst which the most pertinent are: culture’s in-vitro ‘age’ [7–9]; cell seeding densities; media and other growth conditions, which may favour the expansion of only certain MSC subsets [10–12]; and, finally, the donor’s age and possibly gender [6, 13, 14]. In terms of MSC heterogeneity in vivo, it has now become clear that the cells’ tissue and anatomical residence is most important, particularly in terms of MSC differentiation capacity [15–19]. But even in the same tissue, such as BM, is there a biological and physiological basis for the existence of different MSC subsets?

This review will first focus on the in-vivo markers of BM-MSCs in human and mouse species, highlighting common and potentially functionally relevant receptor molecules. The article will then discuss developmental and topographical heterogeneity of MSCs in the BM and the importance of considering donor’s age, gender and health status when studying in-vivo MSC functions in humans. This knowledge could inform novel strategies for prospective isolation of MSCs from their native environments in other tissues. With a better understanding of physiological MSC responses in vivo and their pathological characteristics in diseases such as osteoarthritis (OA) and osteoporosis (OP), MSCs could become future targets for therapeutic interventions.

## Surface markers for prospective isolation of BM-MSCs

The BM was the first tissue from which MSCs were isolated and comprehensively investigated [20, 21]. This compartment is also the prime residence location for another adult stem/progenitor cell; that is, the HSC [22]. The BM is a highly heterogeneous tissue that, in addition to MSCs and HSCs, is composed of their progeny, including fully differentiated cells such as fat cells or plasma cells, as well as of endothelial cells and other non-haematopoietic elements such as nerve endings [23]. Furthermore, BM does not exist in isolation and is intimately connected with surrounding bone. Endosteal (inner bone) surfaces, which are in direct contact with the BM, are covered with ‘lining’ cells that contain MSCs [24], their short-lived (osteoblasts) [25] and long-lived progeny [26] as well as most immature, quiescent HSCs [27]. Whilst in the past the search for BM-MSCs was limited to BM aspirates, more recent findings clearly showed that not all MSCs are obtained by aspiration [24, 28] and that enzymatic digestion of bone is definitely needed to recover additional MSCs from the bone-lining location [28–31].

In cell therapy and tissue engineering communities, MSC isolation commonly implies the production of plastic adherent cultures starting from minimally processed BM aspirates or tissue digests [3]. This method represents a retrospective way of isolating and expanding culture-initiating MSCs whereby contaminating non-MSCs are lost owing to incompatible culture conditions, whereas MSCs are amplified (culture-based selection). In contrast, prospective MSC isolation requires having a candidate marker or markers to purify putative MSC subpopulation(s), followed by their in-vitro expansion and further functional tests such as multipotentiality, immunomodulation or secretion of trophic factors [3, 6, 31, 32] (marker-based selection). Up to now, in-vivo MSC markers suitable for prospective BM-MSC isolation were discovered either by screening of available hybridomas [33–35], from topographical ‘clues’ on histological sections, as was the case for CD271 [36], or from large gene array datasets comparing cultured MSCs with negative control skin fibroblasts or hematopoietic lineage cells [19, 37]. Naturally, markers of cultured MSCs, such as CD73, CD105 and CD90, have also been tested in both human and mouse species, and showed various degrees of success as single markers [3, 31, 38–42] (Table 1). At this point, it is important to note that the role of these various surface markers in MSC physiology in vivo remains largely unknown (Table 1). In fact, the best ‘isolation’ markers could be those which have a minimal role in MSC functionality so that the process of MSC isolation itself has a minimal bearing on possible marker-mediated signal transduction and gene expression in isolated MSCs.

**Table 1:** Markers and potential functions of native BM-MSCs

In-vivo MSC marker	Potential function on MSCs	Host/model	References
CD271	Osteogenesis	Human, murine cell line	[96]
	Interactions with other BM cells	Human	[51, 97]
MSCA-1/Stro-3/non-specific alkaline phosphatase	Osteogenesis/mineralization	Human	[34, 98]
Stro-1	Migration/homing	Human	[99]
CD146	Related to MSC topography, interaction with other BM cells	Human	[24, 50, 54, 100]
Leptin R (CD295)	Controlling bone–fat balance	Mouse	[60]
	Age-related	Human	[64]
PDGFRα (CD140a)	Related to immaturity	Mouse/human	[48]/[65]
	Support HSCs	Mouse/human	[30]
	Early adipogenic commitment	Mouse/human	[101]
PDGFRβ (CD140b)	Proliferation	Human	[61]
CD49a	Receptor for collagen and laminin	Human	[39, 102, 103]
CD106	Migration	Human	[50, 51]
	Interaction with other BM cells		[51]
CD51	Interaction with HSCs	Mouse/human	[30]
CD200	Immunoregulation	Human	[104]
CD90	Interactions with other BM cells	Human	[39]
		Mouse	[51]

*BM* bone marrow, *HSC* hematopoietic stem cell, *MSC* mesenchymal stem/stromal cell, *MSCA-1* mesenchymal stem cell antigen-1, *PDGFR* platelet-derived growth factor receptor

## Classes of BM-MSC surface markers based on their potential functions

In BM aspirates, in flushed contents of cortical bones or, to a lesser extent, in cancellous bone tissue digests, MSCs represent a minority amongst other cell entities [21, 30, 31, 43, 44]. Therefore, so-called ‘negative’ markers are commonly used as the first ‘pre-enrichment’ step in order to enrich MSCs to a certain degree of purity (>1 %) required for subsequent downstream investigations [24, 30]. In particular, the CD45 antigen has been the most commonly used negative selection marker in both human studies [24, 30, 45, 46] and mouse studies [30, 47, 48].

In humans, CD271 and mesenchymal stem cell antigen-1 (MSCA-1; tissue non-specific alkaline phosphatase) have been proposed as specific positive markers for BM-MSCs [34, 40, 49–51]. Stro-1, the first-discovered marker of human BM-MSCs, is cross-reactive with erythroblasts [52] and hence needs to be used in combination with other positive markers [53–55]. Various integrin molecules (CD49a, CD106 and CD146) have been independently validated as expressed on human in-vivo BM-MSCs in numerous original and more recent studies (Table 1). Importantly, the MSC integrin expression pattern seems to be dependent on MSC topographical location; for example, CD146 is expressed on MSCs located perivascularly, but it is absent on MSCs resident in the bone-lining location [24]. Integrins are involved in cell-to-cell and cell-to-matrix interactions [56]. Therefore, a future discovery of more comprehensive patterns of integrin expression on MSCs in different BM niches in the BM could shed more light on their functions and behaviours in vivo. A similar study pertaining to chemokine receptors on BM-MSCs [57–59] could be very valuable in terms of our current understanding of their migration and their homing properties, particularly in relation to fracture repair and bone remodelling processes.

In the mouse system, integrin αV (CD51) [30] has attracted increased attention as being specific for BM-MSCs; however, more recent findings have highlighted the value of growth factor receptors such as platelet-derived growth factor receptor (PDGFR) alpha (CD140a) [30, 48] and leptin receptor (CD295) [60] for the selection of mouse MSCs. These molecules, as well as PDGFRβ (CD140b), have been contemporarily shown to be expressed on human BM-MSCs [60–62]; these surface molecules, in our opinion, therefore represent the first set of common markers applicable to both mouse and human species.

Human Stro-1-positive or CD271-positive BM-MSCs additionally express a large number of other growth factor receptors; for example, epidermal growth factor receptor (EGFR) and insulin-like growth factor receptor (IGFR). Notably, some of these molecules have a clear proliferation-promoting effect on MSCs [63]. In fact, the expression levels of these growth factor receptors on MSCs might indicate the level of their ‘readiness’ to respond to respective growth factor signals [62]. Yet only limited data exist for bone morphogenetic protein (BMP)/transforming growth factor (TGF) beta [64] and Wnt pathway receptor expression [61, 64, 65] on human or mouse MSCs in vivo; studies on these targets are clearly merited given the important role of these pathways in the maintenance and repair of bone [66].

PDGFRα (CD140a), in combination with CD271, has been most recently proposed as a valuable discriminatory marker combination for highly enriched human BM-MSCs, but the data remain controversial. A recent study by Pinho et al. [30] showed that MSCs in fetal human BM feature expression of CD271 plus CD140a. On the other hand, Li et al. [65] recently demonstrated that in adult human BM true highly clonogenic MSCs express CD271 but not CD140a. The authors suggested that CD140a may be developmentally regulated [65], a feature also observed in relation to CD146 expression in fetal, paediatric and adult human BM [67]. In addition to being regulated developmentally, in vivo MSC receptors could possibly be regulated physiologically. For example, our recent study using a cohort of fracture patients has shown that CD140a and CD140b expression on their BM CD271^+^ MSCs was changeable and directly correlated with the levels of PDGFs (as well as platelet levels) in patients’ blood [62]. This observation suggests that MSCs at a site remote to injury might react to systemically driven changes in corresponding signalling molecules. Based on these considerations it might be reasonable to suggest that cytokine and growth factor receptors on MSCs may not be the most valuable tools for MSC isolation because their levels could be developmentally and physiologically controlled. Conversely, they may be very useful for the study of MSC behaviour in vivo, especially with respect to donor age, gender and physiological/disease status.

Furthermore, it is likely that standard (but not yet fully controlled) conditions for growing MSCs in fetal calf serum, autologous serum or with the addition of platelet lysates could in fact select for only those MSCs that have a corresponding set of growth factor receptors and correspondingly ‘de-select’ for receptor-negative cells. For example, culturing MSCs in media containing platelet lysates, rich in human PDGF-BB, could ‘select’ (i.e. induce enhanced proliferation) of MSCs that have high levels of CD140b receptors and de-select for CD140b-negative MSCs. Further causes for apparent differences in the phenotypes between in vivo and cultured MSCs have been elaborated in other previous publications [45, 61, 68, 69].

## BM-MSC heterogeneity: topography, age, gender and disease

As alluded to earlier, cell-to-cell and batch-to-batch heterogeneity of cultured MSCs can in some way reflect the heterogeneity of in vivo MSC populations. MSCs located perivascularly may have a markedly different set of functions compared with bone-lining MSCs. In the bone-lining compartment itself, MSCs are mixed together with their progeny: active osteoblasts [25], which exist only transiently in bone remodelling areas, and quiescent osteoblast descendants predominant in non-remodelling areas [26]. Specific surface markers for these two types of mature MSC-lineage cells have not so far been described in humans, despite some data on their differing transcriptional signatures in the mouse [70]. Stripping off all lining cells from the bone surface with the use of enzyme, as is performed currently [28, 29, 51], is bound to result in mixed mesenchymal-lineage populations that differ in their maturity, which subsequently contributes to cultured MSC heterogeneity.

Age plays a profound role in shaping our skeleton. BM-MSCs are involved in bone remodelling processes directly (as progenitors of osteoblasts) and indirectly (via osteoblast control of osteoclast activation). It can therefore be expected that in-vivo MSC ageing (either in relation to their numbers, function or both) can have a direct bearing on bone physiology in aged adults. Several studies have investigated whether and how MSCs may age in vivo; for example, as a result of telomere shortening processes [8, 29] or via changes to the transcription of Wnt pathway receptor genes [64]. It is important to note that extracellular matrix produced by aged MSCs may further contribute to their ageing [71], suggesting an autocrine mechanism of regulation.

Age-related diseases such as OA and OP are associated with marked changes in bone strength and architecture, and are suggested to involve a defect (or altered function) in patients’ BM-MSCs [72–77]. Owing to the scarcity of healthy human material (BM and bone), it is not surprising that many studies exploring human BM-MSC biology in vivo utilize OA femoral heads [51, 78]. Nevertheless, the effect of disease on these MSCs should not be overlooked. As reported recently using mouse models of OA, the disease process itself is associated with increased subchondral bone MSC numbers and alterations in their intracellular signalling cascades leading to aberrant bone formation and angiogenesis for OA progression [73]. This process may be even more pertinent in the case of OP, in which alterations in MSC numbers and their responsiveness to leptin or to BMPs have been documented [75–77]. The study of growth factor and hormone receptor expression on OP-MSCs could lead to the discovery of novel compounds capable of switching the balance from bone destruction and in favour of bone formation in OP.

Several reports have indicated some gender-related differences in BM-MSCs from humans [6, 79] and other species [14, 80, 81]. Could these also be related to gender differences between MSCs in vivo? The data collected from the Leeds laboratory over the years indicate no significant difference in MSC numbers per millilitre of BM aspirate between age-matched males and females, regardless of whether BM samples were first processed for mononuclear cell isolation or used directly. Interestingly, Seeback et al. [79] documented significantly different BM-MSC responses to skeletal injury between males and females. Caution should be taken when interpreting BM aspirate data, however, because the quality of aspirates in terms of their total MSC numbers is considerably dependent on the surgical aspiration technique and dilution with blood [44, 82, 83], which is significantly variable not only between different institutions but also between different surgeons.

We have reported recently that BM-MSCs from male and female donors express androgen receptor [6], but their responsiveness to sex hormones in general remains underexplored. This knowledge could be potentially exploitable therapeutically; for example, by uncovering sex hormone sensitivity and downstream signalling cascades in BM-MSCs in women with postmenopausal OP.

## MSC heterogeneity: different developmental origins

Human skull and neck bones are well known to be neural-crest derived whereas the remainder of the skeleton is mesoderm derived [84, 85]. Transcriptional differences in MSCs grown from skull and mesoderm-derived bones have been documented previously [15]. Nevertheless, there is no reason to suggest that MSCs in different bones are firmly fixed in their original locations and unable to migrate to other tissues. In fact, mouse BM-MSCs were found recently to represent a mixture of neural-crest and mesoderm-derived cells [86]. In the mouse, MSCs can easily circulate and home to injured tissues [87, 88], whereas in humans this ability seems to be lost, or at least impaired, at birth. MSCs can be readily found in fetal circulation in humans [89], but these cells have been detected only at exceptionally low numbers in adult human peripheral blood, even after a significant physiological insult [62, 88, 90, 91]. The loss of MSC ability to circulate in the blood of humans post-birth could be related to differential expression of some developmentally regulated molecules on their surface (e.g. CD146, CD140a or others), as mentioned in "Classes of BM-MSC surface markers based on their potential functions" above.

Despite the observation that endogenous (not transplanted) human MSCs are unable to be distributed across long distances via the systemic circulation, their short-distance migration between the adjacent tissues remains plausible. MSCs express a broad portfolio of chemokine receptors enabling them to migrate along the chemokine gradients or, in contrast, to be retained in their original places [58]. Based on these considerations, it is possible to suggest that MSCs in any given tissue, including the BM, may represent a mixture of locally derived cells as well as ‘migrants’ from the neighbouring tissues.

## Conclusion

The BM is the tissue in which MSCs were discovered originally and remains the best-studied tissue source of MSCs. Even a quick look at the current state of the art in in-vivo MSCs in human and mouse BM suggests that a single marker specific for all MSC ‘shades and colours’ is unlikely to be found soon. In-vivo BM-MSC heterogeneity could be explored in the future using different approaches. For example, using a combination of immunohistochemical and cell sorting techniques, separate BM-MSC subsets can be isolated based on their topographical residence. Specific molecular marks indicative of MSC embryonic tissue origins, such as HOX and other ‘positional identity’ genes [17, 92], could be used next to shed a light on migratory routes of different classes of MSCs during development and in early childhood. Equally, their differential survival or impaired function during ageing could help to better understand the role of BM-MSCs in the development of age-related bone diseases such as OP.

When BM-MSCs from different species are compared, several considerations should be taken into account, the prime one being the type of host bone used to extract the MSCs. Most BM-MSC investigations in mice have been performed using flushed contents of cortical bones, whereas human BM-MSC research has primarily explored MSCs resident in marrow spaces inside certain cancellous bones (most often, the iliac crest). Only limited data yet exist on the gene expression profiles of uncultured BM-MSCs from donor-matched cancellous and cortical bones in humans [93]. Even if the same type of bone (e.g. femur) is used in human and mouse research, it is important to consider the effects of different mechanical loads experienced by bipeds as opposed to quadrupeds; the mechanical effects driving bone remodelling and hence the physiological demand on femoral MSCs in bipeds are likely to be very unique. Still, it is very encouraging to observe some emerging commonality in CD140 and CD295 receptor expression on BM-MSCs from both mouse and human species (Table 1). These common receptor molecules may be indicative of key BM-MSC functions distinct from their mechanically driven bone-remodelling activity; for example, of their control of the bone–fat balance in the marrow or of their support to HSCs.

Finally, to what extent can BM-MSC knowledge be extrapolated to MSCs in other issues? In our view, the best ‘toolkit’ to isolate the bulk of tissue-resident MSCs may not overlap with BM-MSCs [18], and this needs to be looked into on a tissue-to-tissue basis. Potential back and forth ‘passaging’ of MSCs between the neighbouring tissues should be also considered, which could explain, at least in part, the observed heterogeneity of MSCs in the respective tissue.

Most MSCs are lodged within the stroma of solid tissues and organs, making it very difficult to study their self-renewal and their participation in physiological tissue renewal in a classical manner similar to HSCs or even BM-MSCs [94]. In this respect, gene-tracing experiments in mouse models are of a paramount importance in order to understand normal MSC behaviour in tissues and organs and their responses to injury or disease [95]. With further appreciation of probable differences in MSC biology between ‘mice and men’, this future knowledge is likely to generate new ideas and bring forward new treatments for many human diseases.

## Note

This article is part of a thematic series ‘*Mesenchymal Stem/Stromal Cells*—An update’. Other articles in this series can be found at http://www.biomedcentral.com/series/mesenchymal

## Abbreviations

BM: Bone marrow
BMP: Bone morphogenetic protein
EGFR: Epidermal growth factor receptor
HSC: Hematopoietic stem cell
IGFR: Insulin-like growth factor receptor
MSC: Mesenchymal stem/stromal cell
OA: Osteoarthritis
OP: Osteoporosis
PDGFR: Platelet-derived growth factor receptor
TGF: Transforming growth factor
